# Contemporary Patterns of Care for Low-Grade Glioma in Australia and New Zealand

**DOI:** 10.3390/curroncol32030183

**Published:** 2025-03-20

**Authors:** Meghana Maddula, Nicholas McNamee, Hui K. Gan, Laveniya Satgunaseelan, Eng-Siew Koh, Catherine H. Han, Subotheni Thavaneswaran

**Affiliations:** 1 The Kinghorn Cancer Centre, St. Vincent’s Hospital, 370 Victoria St., Darlinghurst, NSW 2010, Australia; nicholas.mcnamee@svha.org.au; 2 Faculty of Medicine and Health, University of New South Wales, Sydney, NSW 2052, Australia; 3 Austin Health, Austin Hospital, Heidelberg, VIC 3084, Australia; hui.gan@austin.org.au; 4 Cancer Therapies and Biology Group, Centre of Research Excellence in Brain Tumours, Olivia Newton-John Cancer Wellness and Research Centre, Austin Hospital, Heidelberg, VIC 3084, Australia; 5 School of Cancer Medicine, La Trobe University, Heidelberg, VIC 3079, Australia; 6 Department of Neuropathology, Royal Prince Alfred Hospital, Sydney, NSW 2050, Australia; laveniya.satgunaseelan@health.nsw.gov.au; 7 Department of Radiation Oncology, Liverpool Hospital, Sydney, NSW 2170, Australia; engsiew.koh@health.nsw.gov.au; 8 School of Clinical Medicine, South West Sydney Clinical Campuses, Faculty of Medicine and Health, University of New South Wales, Liverpool, NSW 2170, Australia; 9 Auckland Oncology & Auckland City Hospital, Auckland 1023, New Zealand; c.han@auckland.ac.nz; 10 Department of Pharmacology and Clinical Pharmacology, Faculty of Medical and Health Sciences University of Auckland, Auckland 1023, New Zealand; 11 NHMRC Clinical Trials Centre, University of Sydney, Sydney, NSW 2050, Australia

**Keywords:** low grade glioma, astrocytoma, oligodendroglioma, vorasidenib, IDH inhibitors, chemotherapy, radiotherapy

## Abstract

Aim: The management of low-grade gliomas (LGGs) is evolving with new insights into disease biology. Furthermore, recently, the phase III INDIGO^1^ study highlighted the benefits of an IDH inhibitor, vorasidenib, in treating residual or recurrent grade 2 IDH-mutant gliomas following surgery alone. We aimed to characterise the current patterns of care for patients with LGGs in Australia and New Zealand, including the role of vorasidenib. Methods: An online survey examining respondents’ practice setting, caseload, and preferred treatment approach to three clinical scenarios was distributed through the Cooperative Trials Group for Neuro-Oncology, New Zealand Aotearoa Neuro-Oncology Society, and the Australian and New Zealand Society for Neuropathology in December 2023 with three reminders in April, June, and September of 2024. Results: The survey response rate was 19.6% (57/291), 87.7% from Australia, and 12.3% from New Zealand, spanning medical oncology (45.7%), pathology (22.8%), radiation oncology (17.5%), and neurosurgery (14.0%). Case 1 examined an IDH-mutant grade 2 astrocytoma following gross total resection. Observation alone was recommended by 93%. Case 2 examined an incompletely resected IDH-mutant grade 2 astrocytoma. If feasible, 38% recommended further surgery and 83% adjuvant chemotherapy and radiotherapy. After 12 months of disease stability, 53% of the respondents preferred vorasidenib over the existing therapies. Case 3 examined an incompletely resected IDH-mutant grade 3 oligodendroglioma. No respondents recommended observation alone, with 26% recommending salvage surgery and 97% recommending further chemotherapy and radiotherapy. Conclusions: This study describes current management practices for LGGs in Australia and New Zealand, showing ongoing variation and a cautious approach to integrating IDH inhibitors. This highlights the critical role of multidisciplinary team-based decision-making in increasingly complex clinical situations.

## 1. Background

Low-grade gliomas (LGGs) are a heterogeneous group of primary brain tumours, typically characterised by their slower growth compared to high-grade gliomas [1,2]. Despite their more indolent nature initially, LGGs pose significant challenges in management, particularly in the recurrent setting where they may behave unpredictably. The cornerstone of management of LGGs remains cytoreductive surgery, with the aim of achieving a gross total resection as the extent of resection impacts survival outcomes [3,4]. For patients with grade 2 gliomas, considered ‘low risk’ based on young age (<40 years), smaller tumour size, and gross total resection, active surveillance with regular surveillance MRI scans may be sufficient [5]. However, in the presence of residual tumour or older age (>40 years), adjuvant therapies comprise radiotherapy and chemotherapy. The evidence base supporting radiotherapy in this setting includes several key studies, the EORTC 22033 and EORTC 22845, which have shown improvements in progression-free survival [6,7]. More recently, chemotherapy, particularly with temozolomide or procarbazine, lomustine, and vincristine (PCV), has been shown to improve survival outcomes when combined with radiotherapy in high-risk patients, as demonstrated in the RTOG 9802 study [8,9].

*IDH1/2* mutations are an established biomarker in the classification and prognostication of LGGs [10,11,12]. More recently, other molecular biomarkers, such as *CDKN2A/B* homozygous deletions, have been shown to alter the biology of these low-grade tumours [13,14]. In fact, this molecular feature upgrades a histologically low-grade IDH-mutant glioma (grade 2 by histology) to a high-grade glioma (grade 3 or 4), according to the current World Health Organisation Classification of Tumours of the Central Nervous System (5th edition, 2021) (CNS WHO5) [1]. However, the ability to target molecular alterations therapeutically in gliomas has been limited until recently. The phase III INDIGO study demonstrated the efficacy of an IDH inhibitor, vorasidenib, in patients with residual or recurrent grade 2 IDH-mutant gliomas after surgery alone [15]. Vorasidenib significantly improved progression-free survival compared to placebo, providing a promising new treatment for a subset of LGG patients who were considered appropriate for observation alone for at least 12 months after surgery and not requiring immediate adjuvant therapy.

The RTOG 9802 trial [16] demonstrated improved survival in high-risk low-grade gliomas (LGG) with the addition of adjuvant PCV chemotherapy to radiotherapy (mOS 13.3 vs. 7.8 years). Despite this, a COGNO survey revealed variability in clinician preferences for adjuvant therapy, with postoperative observation remaining common for lower-risk cases and temozolomide favoured over PCV by 81% but underutilised in practice. A decade from this study and an evolving treatment landscape of LGGs prompted our study [16]. We aimed to better understand the proposed integration of this therapy into the complex management algorithm and clinical care of patients with LGGs.

## 2. Methods

This was a cross-sectional survey designed to assess current management practices for LGGs among relevant clinicians in Australia and New Zealand (ANZ). The target population was currently clinically active neuro-/medical oncologists, radiation oncologists, neurosurgeons, and neuropathologists involved in the care of patients with LGG as part of their usual practice. The survey included questions addressing respondent practice setting, case volume, molecular testing practices, and clinical decision-making in three specific hypothetical scenarios involving IDH-mutant LGG patients (File S1).

The online survey using *SurveyMethods* was initially distributed in December 2023, with a further three reminders sent in 2024 to members of the Cooperative Trials Group for Neuro-Oncology (COGNO), the New Zealand Aotearoa Neuro-Oncology Society (NANOS), and the Australian and New Zealand Society for Neuropathology (ANZSNP). Targeted reminders regarding survey completion were also sent to key clinicians at centres managing low-grade glioma patients across ANZ.

Data were analysed using descriptive statistics to identify patterns and differences in treatment strategies based on three clinical scenarios presented, outlined below. The response rate denominator included currently clinically active recipients only.

**Scenario 1**

A 38-year-old female, gross total resection of an IDH-mutant grade 2 astrocytoma

No residual enhancing disease remaining on immediate postoperative MRI (<72 h)

Molecular profile: pending

Clinically asymptomatic (stable seizures on levetiracetam), prompt surgical recovery.

**Scenario 2**

A 58-year-old male with newly diagnosed grade 2 astrocytoma.

Subtotal resection: the non-enhancing rim of residual disease on postoperative MRI T2 FLAIR sequences.

Clinically recovering well postoperatively and asymptomatic.

Molecular profile: *IDH1* mutation detected, 1p/19q codeletion NOT detected, *TP53* variant detected, *CDKN2A/B* homozygous deletion NOT detected.

**Scenario 3**

A 58-year-old female with newly diagnosed IDH-mutant grade 3 oligodendroglioma.

Near gross total resection with both enhancing and non-enhancing residual disease on postoperative MRI. Clinically symptomatic.

Molecular profile: *IDH1* variant detected, *TERT* promoter variant detected, 1p/19q codeletion detected

## 3. Results

### 3.1. Characteristics of Respondents

**Figure 1:**
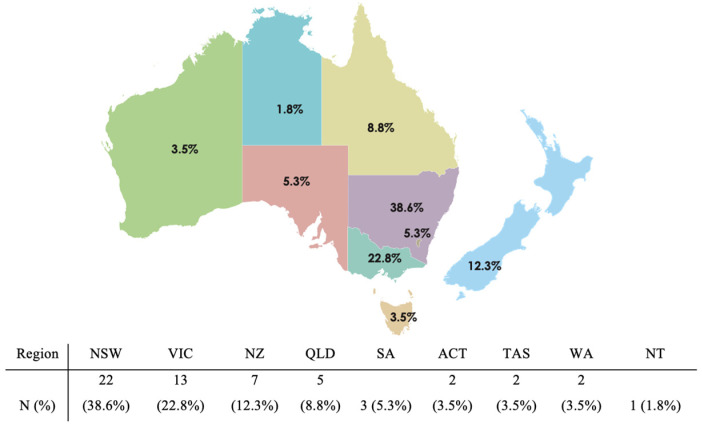
Distribution of survey respondents across Australia and New Zealand.

There was a total of 57 responses with a response rate of 20% (n = 57/291). As shown in Table 1, respondents represented medical oncology (46%), pathology (23%), radiation oncology (18%), and neurosurgery (14%) specialty [Table 1]. Practice settings were primarily metropolitan, with a smaller proportion of practice settings being regional or remote centres. There were respondents from all Australian states and territories (NSW = 39%, VIC = 23%, QLD = 9%, SA = 5%, TAS = 4%, ACT = 4%, WA = 4%, NT = 2%), as well as New Zealand (12%) [Figure 1]. The majority (n = 52/54, 96%) of respondents participate in neuro-oncology multidisciplinary team (MDT) meetings. Most participants completed the survey alone (n = 43/57, 75%), with 13 (22.8%) respondents completing the survey with at least 1 of their MDT colleagues.

### 3.2. Volume and Scope of Practice

Respondents reported varying case volumes at their centre across glioma subtypes, with 72% of respondents providing these numbers based on an estimate only versus 9% based on a review of institutional data [Table 2]. Centres reported the highest case volume for grade 4 astrocytomas (mean 36.0 cases/year, IQR 10–50), with the lowest for grade 3 oligodendrogliomas (mean 4.6 cases/year, IQR 2–6). Clinical trial participation was limited across all tumour types, with the highest mean enrolment for grade 4 astrocytomas (6.2 patients/year, IQR 0–8).

### 3.3. Molecular Testing

Molecular testing practices varied across respondents, with a total of 54 clinicians detailing specific molecular testing they would request at initial diagnosis. Among these respondents, 52% consistently requested 1p/19q codeletion status, and 29% routinely requested *CDKN2A/B* deletion status. Despite diagnostic, prognostic, and therapeutic implications for many of the listed genomic biomarkers listed (File S2, a proportion of respondents would never request them; 1p/19q codeletion (2%), *MGMT* methylation (4%), *BRAF*, H3 K27M, *EGFR* (8%), and *CDKN2A/B* (10%).

Of the five respondents who reported ‘never’ testing for *CDKN2A/B* deletion, all worked in public institutions, with four based in metropolitan centres and one in a regional or rural location.

## 4. INDIGO Study

The survey examined respondents’ familiarity with the INDIGO trial [15] and the potential incorporation of vorasidenib into clinical practice. Among the respondents, 73% stated they were familiar with the INDIGO trial findings. When asked about vorasidenib’s role, respondents indicated they would consider it suitable for an average of 41% (IQR 26–50%) of grade 2 glioma patients.

Clinicians showed caution in applying vorasidenib to grade 3 cases, given the limited evidence from the INDIGO trial, which focused specifically on grade 2 IDH-mutant gliomas. Clinicians reported they would consider vorasidenib in 23% (IQR 5–25%) of grade 3 glioma patients. Some respondents emphasised the need for longer follow-up and additional comparative studies to fully determine vorasidenib’s role relative to current standard-of-care therapies, particularly in the management of higher-grade gliomas.

## 5. Clinical Scenarios

### 5.1. Scenario 1

**Figure 2:**
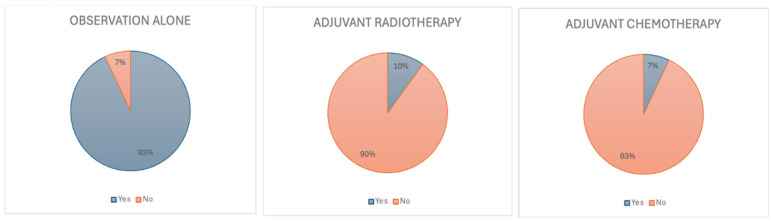
Scenario 1. Initial recommendation for management.

In the case of a 38-year-old female with a completely resected IDH-mutant grade 2 astrocytoma, the majority of clinicians (93%) recommended observation alone as the preferred management strategy [Figure 2]. Among these, 63% advocated for MRI surveillance every three months initially, with most suggesting an extension to six-monthly intervals after a period of demonstrated clinical stability. Amongst 22 medical oncologists who responded to this question, 95% recommended observation alone. Amongst eight radiation oncologists who responded to this question, 88% also recommended observation alone.

**Figure 3:**
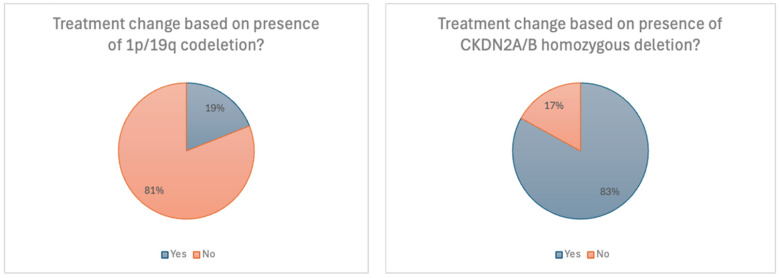
Scenario 1. Treatment recommendations based on specific molecular findings.

When presented with specific molecular findings, respondents’ management strategies varied. In the event that the primary tumour pathology revealed a 1p/19q codeletion confirming a diagnosis of grade 2 oligodendroglioma, 19% of clinicians indicated they would change their management approach. Of these (n = 8), 5 recommended adjuvant chemotherapy in the presence of a 1p/19q codeletion [Figure 3]. In contrast to this, the presence of a *CDKN2A/B* homozygous deletion prompted 87% of clinicians to classify the tumour as high-grade and pursue adjuvant therapy accordingly. Of 22 medical oncologists who responded to this question, 86% recommended the same. We interpret this response to reflect the adverse prognostic importance of *CDKN2A/B* homozygous deletion in driving treatment escalation [Figure 3].

The survey also explored clinicians’ considerations in selecting observation, adjuvant radiotherapy, or adjuvant chemotherapy [Table 3]. The volume of residual disease emerged as a key factor influencing decisions across all treatment options. For those favouring observation, a residual disease volume of less than 2 cm was the primary consideration, followed by patient age (<40 years) and favourable tumour molecular profiles. Conversely, for adjuvant radiotherapy and chemotherapy, a residual disease volume of ≥2 cm, patient age ≥ 40 years, and tumour molecular characteristics were the leading factors guiding treatment choices.

**Figure 4:**
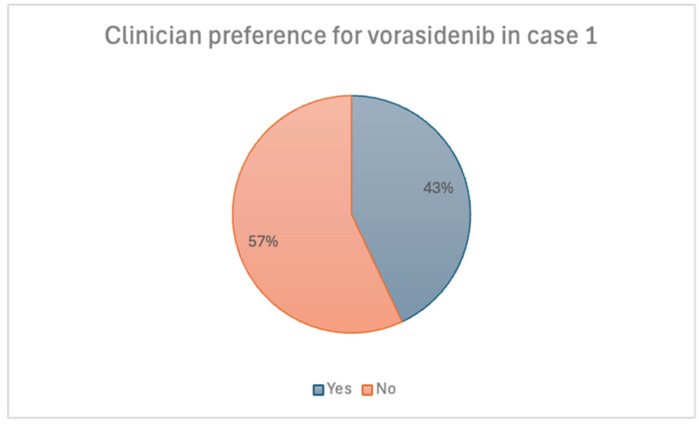
Scenario 1. Preference for vorasidenib.

Clinicians were also asked whether they would consider vorasidenib over current adjuvant therapies if it was available. Among the 37 respondents, 57% indicated they would not prefer vorasidenib in this case [Figure 4]. When evaluating responses by sub-specialty, 62% of medical oncologists and 83% of radiation oncologists did not prefer vorasidenib. Those who favoured standard therapy options attributed this to a lack of supporting data for vorasidenib use in patients without residual disease, along with a preference to reserve vorasidenib for use upon recurrence.

**Figure 5:**
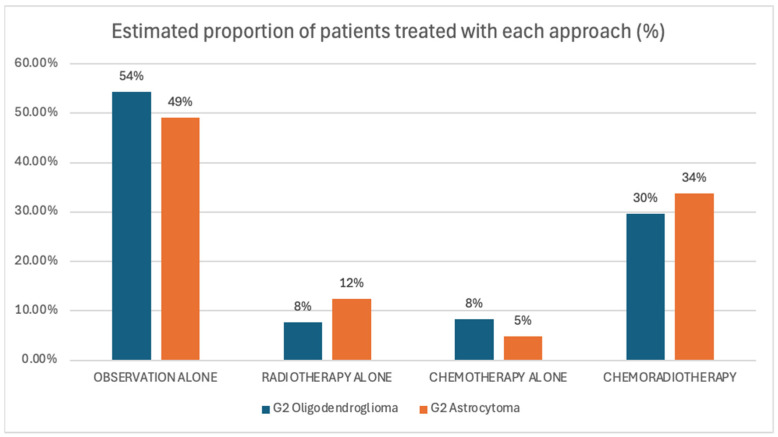
Scenario 1. Recommended initial treatment approaches.

When asked for initial management approaches for grade 2 gliomas, observation alone was the most frequent recommendation amongst 54% of respondents for oligodendrogliomas and 49% for astrocytomas [Figure 5]. Following observation, the next most frequent recommendation was a combination of chemotherapy and radiotherapy, with 33% selecting this approach for astrocytomas and 30% for oligodendrogliomas. Single-modality treatments, such as radiotherapy alone or chemotherapy alone, were less commonly chosen.

**Figure 6:**
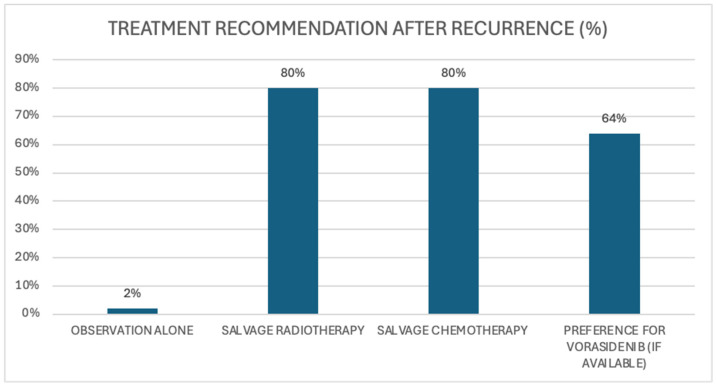
Scenario 1. Recommended approach for treatment approach at disease recurrence.

Upon disease recurrence in our scenario 1 patient after 24 months of observation and further surgical resection, 80% of the 42 clinicians who responded preferred adjuvant combination therapy with chemotherapy and radiotherapy in the absence of vorasidenib availability [Figure 6]. Among the 21 clinicians who specified a choice of chemotherapy agent, 17 (81%) advocated for the use of temozolomide. In this same context, if vorasidenib were available at the time of recurrence, 64% of clinicians indicated they would prefer this over traditional salvage therapies; this included 68% of medical oncologists and 4% of radiation oncologists. Notably, 88% of those who favoured vorasidenib had initially recommended observation alone in alignment with the INDIGO trial population. Among the clinicians who did not advocate for vorasidenib, common reasons included the lack of direct comparative data with existing standard-of-care therapies and uncertainty regarding longer-term outcomes. Of note, however, was some ambiguity in this survey question, as it did not clarify whether residual disease was evident after the re-resection.

### 5.2. Scenario 2

**Figure 7:**
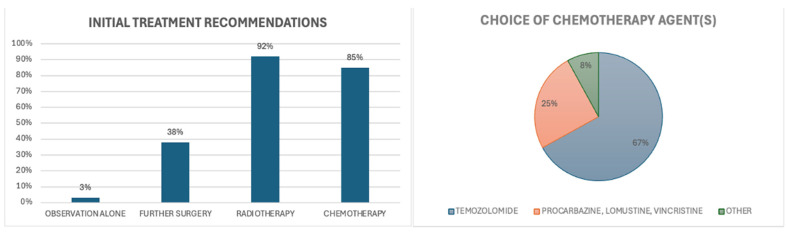
Scenario 2. Initial recommendation for management.

In the case of a 58-year-old male with a newly diagnosed IDH-mutant grade 2 astrocytoma and subtotal resection, the majority of clinicians, including 96% of medical oncologists, 100% of radiation oncologists and 67% of surgeons, recommended additional treatment [Figure 7]. Of 39 clinicians who responded to this question, only one suggested observation alone. Over one-third (38%) recommended salvage surgery if feasible, and the majority (83%) recommended a combination of adjuvant chemotherapy and radiotherapy. Among those who specified a chemotherapy regimen (n = 25), temozolomide was the preferred agent, selected by 64% of respondents [Figure 7].

**Figure 8:**
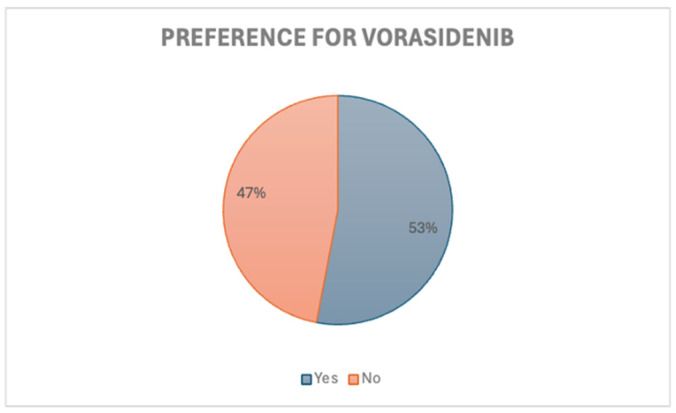
Scenario 2. Preference for vorasidenib.

When asked if vorasidenib would be preferred over traditional adjuvant therapies after a 12-month period of stable disease, responses were divided, with 53% indicating that they would consider vorasidenib in this scenario [Figure 8]. This included 59% of medical oncologists and 85% of radiation oncologists. As per the INDIGO study, patients with residual or recurrent grade 2 IDH-mutant glioma who had no prior treatment other than surgery were eligible for vorasidenib. This cohort of patients would, therefore, be eligible if they had not received prior adjuvant therapies. However, it is important to consider here that most respondents recommended adjuvant chemotherapy and radiation upfront based on high-risk features of residual disease and older age over 40 years.

Among those who did not recommend vorasidenib in this setting, 50% were medical oncologists, 33% radiation oncologists, 10% neurosurgeons, and 7% pathologists, with 72% of this group stating familiarity with the INDIGO findings at the time of survey completion. The key concerns included the lack of direct comparative data with existing therapies and the need for extended follow-up to confirm the progression-free survival benefit observed in INDIGO.

These findings highlight some of the challenges in applying the INDIGO trial results in clinical practice. Specifically, the requirement for a 12-month stability period before initiating vorasidenib diverges from current standard-of-care oncological approaches, which typically favour the immediate intensification of treatment for patients with residual disease. Additionally, an adjuvant treatment decision made after initial surgery would technically preclude the later use of vorasidenib. Clinicians may perceive delaying immediate adjuvant treatment with radiotherapy and chemotherapy in a ‘high-risk’ patient as an unnecessary risk, given the lack of direct comparative data with vorasidenib.

### 5.3. Scenario 3

**Figure 9:**
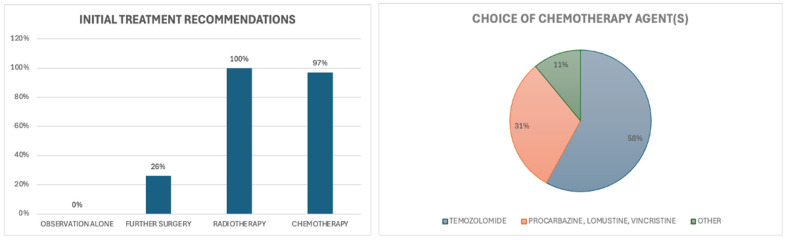
Scenario 3. Initial recommendations for management.

In the case of a 58-year-old female with a newly diagnosed IDH-mutant grade 3 oligodendroglioma, with both enhancing and non-enhancing residual disease visible on postoperative MRI after a near-total resection, there was a strong consensus among clinicians in favour of further treatment beyond observation [Figure 9]. All medical oncologists, radiation oncologists, and surgeons who responded to this question advocated for further treatment. This question was answered by 38 survey respondents, and none recommended observation alone for this incompletely resected tumour.

Over one-quarter (26%) of clinicians recommended salvage surgery if feasible. The majority (97%) also recommended adjuvant chemotherapy and radiotherapy. Of those who specified a preferred chemotherapy regimen (n = 26), temozolomide was the most frequently chosen option, recommended by the majority (58%) of clinicians [Figure 9].

**Figure 10:**
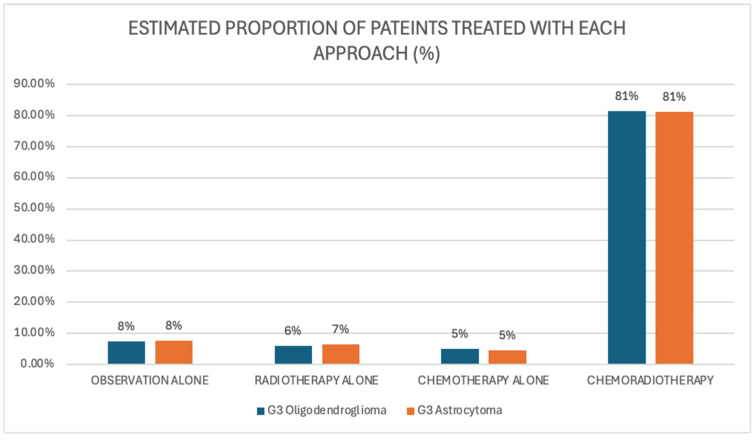
Scenario 3. Recommended initial treatment approaches.

Further analysis of treatment preferences for grade 3 gliomas indicated a strong consensus among clinicians for a combined approach of chemotherapy and radiotherapy for both grade 3 oligodendrogliomas and grade 3 astrocytomas, with over 80% of respondents selecting this treatment strategy in each case [Figure 10]. Single-modality treatment was rarely selected, with only 5% of clinicians recommending chemotherapy alone and 7% choosing radiotherapy alone in this context. Observation without further intervention was recommended by only a small proportion of clinicians: 8% for grade 3 oligodendrogliomas and 8% for grade 3 astrocytomas.

## 6. Discussion

This contemporary pattern of care study, conducted from late 2023 to 2024, provides valuable insights into the current management practices for patients with LGG among clinicians in Australia and New Zealand. It highlights evolving approaches, particularly with the availability of new therapies such as the IDH inhibitor vorasidenib. The findings reveal a cautious and evolving approach to the uptake and incorporation of vorasidenib into clinical care.

We intentionally targeted clinicians and societies directly involved in the management of LGGs to ensure that survey responses reflected expertise in the nuances of evolving classification criteria and treatment landscape. While distribution to a broader group of clinicians may have increased response numbers, it may have introduced variability in familiarity with these factors, potentially limiting the relevance of these findings. While we acknowledge that neuropathologists are not directly involved in the prescription of therapies, they were included for the critical role they play in diagnosing and classifying LGGs and their significant contributions to multidisciplinary discussions that eventually inform treatment recommendations.

We also recognise that there is variability in the number of responses between states and territories across Australia and New Zealand, with the majority of responses being from New South Wales (39%) and Victoria (23%). We believe that this variation primarily reflects the differential distribution of the population and the location of major referral centres rather than a true disparity in interest or management practices across regions. Data from the Australian Bureau of Statistics (June 2024 release) show that over 31% of the Australian population resides in New South Wales and more than 25% in Victoria [17]. This demographic distribution likely explains the observed variation in survey responses, population density, and the concentration of neuro-oncology services.

Key findings from case-based scenarios include a strong preference for observation and surveillance in cases of completely resected IDH-mutant grade 2 gliomas, with a notable shift towards standard salvage therapy and consideration of vorasidenib at recurrence. In cases of incompletely resected grade 2 gliomas, the respondents’ recommendation was for adjuvant chemotherapy and radiotherapy and consideration of vorasidenib in the setting of disease stability. However, there is uncertainty regarding the use of vorasidenib for patients who recur after receipt of standard adjuvant chemo-radiation and exactly which factors impact clinicians’ preference for radiotherapy and chemotherapy over vorasidenib in the upfront setting. Respondents expressed the need for long-term survival data from the INDIGO trial [15] cohort to apply to the relevant subset of patients with LGGs but also acknowledged unanswerable questions amongst patients who did not meet the exact INDIGO trial eligibility. For grade 3 IDH-mutant gliomas, respondents recommended adjuvant radiotherapy and chemotherapy. There remains some uncertainty regarding how emerging therapies, such as IDH inhibitors, can be effectively incorporated alongside existing treatment options—particularly given the limited real-world data available. Kamson et al. recently investigated the real-world experience of glioma response to IDH inhibitors, although ivosidenib rather than vorasidenib, and reported promising results [18]. Their study retrospectively evaluated treatment-naïve patients with IDH1-mutant, non-enhancing, radiologically active grade 2/3 gliomas who subsequently received ivosidenib. The treatment was well-tolerated, and pre-and post-treatment MRI analysis demonstrated a high volumetric response rate [18]. Targeted educational efforts could support clinicians in determining the optimal timing for IDH inhibitor initiation and its integration within established treatment pathways for LGGs.

CNS WHO 5 introduced critical updates that affect how we interpret glioma trials, including INDIGO. The INDIGO trial selected patients based on the 2016 WHO criteria, which primarily used histology for grading [15]. According to the 2021 updates, molecular characteristics such as *CDKN2A/B* homozygous deletion now play a major role, leading to the reclassification of a subset of IDH-mutant low-grade gliomas as grade 4 tumours, reflecting a more aggressive biological profile [1]. This shift implies that some patients previously deemed low-grade in the INDIGO trial may now be categorised as high-grade, impacting prognosis and treatment recommendations. While a detailed breakdown of *CDKN2A/B* copy number status in INDIGO is pending, this study carefully deselected patients with more aggressive tumours by requiring a minimum 12-month observation period following their last surgery. As these updated classification guidelines influence clinical practice, reevaluating patients’ eligibility for targeted treatments like vorasidenib will be essential. Of the five respondents who reported ‘never’ testing for *CDKN2A/B* deletion, all worked in public institutions, with four based in metropolitan centres and 1 in a regional or rural location. This pattern may reflect resource limitations or differing institutional adoption of CNS WHO5. The group included one medical oncologist, two radiation oncologists, one neurosurgeon, and one pathologist.

The strength of this contemporary patterns of care survey is its reach across key organisations in ANZ—COGNO, NANOS and the ANZSNP and targeted centres and MDTs managing LGG patients across ANZ and those with clinical trial capacity for this cohort of patients. It also stands over a decade from the last COGNO-led evaluation of patterns of care in the low-grade glioma space led by K. M. Field et al. [16]. Importantly, respondents spanned all Australian states and territories, metropolitan and regional/remote settings, as well as some respondents whose practice included paediatric and young adult patients. It was also a strength to include respondents and practices from New Zealand. All major medical disciplines providing key input into the management of LGG have been included. Furthermore, almost 90% of respondents were connected to a neuro-oncology MDT, signifying that respondents reflect those with neuro-oncology expertise and actually lead the management of patients with this rare disease. Furthermore, to our knowledge, this is the most recent survey assessing current management practices for LGGs in Australia and New Zealand, conducted in the context of an evolving treatment landscape where IDH inhibitors are now becoming available.

While we acknowledge the modest overall response rate, we believe that the respondents are representative of the key centres and specialists with expertise in the area of LGG, who are actually at the frontline of managing these patients, including high and low volume as well as metropolitan and regional centres. Another limitation was the ambiguity in the hypothetical clinical scenarios presented and the variance in how these may have been interpreted, which in turn may have influenced the recommended management strategies. Despite these limitations, this study provides valuable real-world contemporary data that can guide clinical practice and inform decision-making as vorasidenib becomes available for use in Australia and expectantly to follow in New Zealand.

Future recommendations include targeted educational opportunities regarding the INDIGO trial findings, with specific discussions as to how trial findings should be applied to patients in clinical practice, particularly now that vorasidenib has TGA approval for use in Australia as of 12 September 2024.

## 7. Conclusions

This study provides insight into current management practices for LGGs in Australia and New Zealand and is, importantly, representative of the majority of clinicians involved in LGG treatment and multidisciplinary teams, providing valuable perspectives on current and evolving practice patterns. It highlights ongoing variation in clinical approaches and a cautious approach to integrating IDH inhibitors and emphasises the critical role of multidisciplinary team-based decision-making. The survey results demonstrated a strong preference for observation following a gross total resection of IDH-mutant grade 2 astrocytomas (93%), while an incompletely resected tumour prompted recommendations for further surgery (38%) and adjuvant therapy (83%). In the context of stable disease after 12 months, 53% of respondents preferred vorasidenib over conventional adjuvant therapies, reflecting interest but judicious incorporation of vorasidenib into the current treatment landscape. For IDH-mutant grade 3 oligodendrogliomas, there was consensus on the need for further treatment, with 97% recommending chemotherapy and radiotherapy. Given the evolving role of targeted therapies and molecular classification in LGG management, it will be important to reassess patterns of care in the future to understand the value of IDH inhibitors and their incorporation into standard practice.

## Figures and Tables

**Table 1:** Specialty and practice setting of respondents across Australia and New Zealand.

Specialty, N (%)		Practice Setting ^#^, N (%)	
Medical Oncologist	25 (43.9%)	Metropolitan centre, public	52 (91.2%)
Anatomical Pathologist	13 (22.8%)	Metropolitan centre, private	4 (7.0%)
Radiation Oncologist	10 (17.5%)	Regional or rural centre, public	3 (5.3%)
Neurosurgeon	8 (14.0%)	Paediatric centre	2 (3.5%)
Other	1 * (1.8%)	Regional or rural centre, private	1 (1.8%)

* Paediatric/Adolescent and Young Adult Medical Oncologist ^#^ More than one answer allowed for each respondent.

**Table 2:** Case volume and clinical trial participation.

Tumour Type	Estimate of Cases/Year at Centre, Mean (IQR)	Estimate of Cases Enrolled in Clinical Trial/Year at Centre, Mean (IQR)
Oligodendroglioma grade 2	5.2 (2–6.25)	0.6 (0–0)
Oligodendroglioma grade 3	4.6 (2–6)	0.7 (0–0)
Astrocytoma grade 2	8.8 (3–10)	1.1 (0–0)
Astrocytoma grade 3	7.8 (4–10)	0.7 (0–0)
Astrocytoma grade 4	36.0 (10–50)	6.2 (0–8)

IQR: Interquartile Range.

**Table 3:** Scenario 1. Top factors considered when determining suitability for each treatment option (ranked).

	Observation (Question 21)	Adjuvant Radiotherapy (Question 23)	Adjuvant Chemotherapy (Question 25)
1	Volume of residual disease < 2 cm	Volume of residual disease ≥ 2 cm	Volume of residual disease ≥ 2 cm
2	Age < 40 years old/tumour molecular profile	Age ≥ 40 years old	Tumour molecular profile
3	Patient preference	High symptomatic burden/tumour molecular profile	Age ≥ 40 years old
4	Low symptomatic burden	Patient preference	High symptomatic burden
5	Good performance status	Poor performance status	Patient preference

## Data Availability

Our study data can be accessed by contacting the corresponding author.

## Supplementary Materials

The following supporting information can be downloaded at: https://www.mdpi.com/article/10.3390/curroncol32030183/s1, File S1: Survey; File S2: Genomic and Molecular biomarkers tested.
