# Supplementary material for: Contemporary Patterns of Care for Low-Grade Glioma in Australia and New Zealand
Source: Curr Oncol. 2025 Mar 20;32(3):183. doi: 10.3390/curroncol32030183 (PMC11941661; doi:10.3390/curroncol32030183)
Supplement: Supplementary file 1 [file curroncol-32-00183-s001.zip › curroncol-3507713-supplementary.pdf]

Current Oncology MDPI

Supplementary Materials

File S1. Survey

1. What is your discipline?

Medical Oncologist / Neuro-Oncologist Radiation Oncologist

Neurosurgeon

Pathologist

If other, please specify

____

2. **In which Australian state or territory (or NZ) do you practice?**

3. **Does your centre participate in a Neuro-Oncology multi-disciplinary meeting (MDM/MDT)?** Yes No

4. **Which best describes the type of treatment centre at which you discuss low grade glioma cases? This may be where your MDT is based.**

Metropolitan Centre (public) Regional or Rural Centre (public) Metropolitan Centre (private) Regional or Rural Centre (private) Paediatric/AYA centre

If other, please specify ______________________________

5. **What other specialties in addition to yourself participated in completion of this survey?**

None - just me

Medical Oncologist / Neuro-Oncologist

Radiation Oncologist

Neurosurgeon

Pathologist

If other, please specify

____

6. **How many of the following specialists service your centre?**

Number of specialists

Radiation Oncologists ________________

Pathologists ________________

Care Coordinator ________________

Neurosurgeons ________________

Medical Oncologists / Neuro-Oncologists ________________

Neuro-Specialist Nurse ________________

Clinical Trial Staff ________________

7. **How many new diagnoses of glioma (based on WHO 2021 classification) have been diagnosed at your centre in the past 12 months?**

Oligodendroglioma - grade 2 ________________________

Oligodendroglioma - grade 3 ________________________

Astrocytoma - grade 3 ________________________

Astrocytoma - grade 2 ________________________

Astrocytoma - grade 4 ________________________

8. **Is the answer to the above question 7 based on an estimate or a review of institutional data?**

Estimate only

Review of institutional data

If other, please specify

____

9. **Approximately how many of your patients with the following types of glioma (newly diagnosed or recurrent) are currently enrolled in clinical trials (any phase)?**

Please specify an approximate number of patients.

Oligodendroglioma - grade 2 ________________________

Oligodendroglioma - grade 3 ___________________________

Astrocytoma - grade 3 ___________________________

Astrocytoma - grade 2 ___________________________

Astrocytoma - grade 4 ___________________________

10. **What molecular profiling is routinely requested for patients with newly diagnosed glioma at the time of initial surgery or biopsy at your centre?**

IDH1 (R132H only) = Always Never Sometimes

IDH1 & IDH2 = Always Never Sometimes

CDKN2A/B homozygous deletion H3 K27 status = Always Never Sometimes

EGFR amplification = Always Never Sometimes

1p/19q codeletion = Always Never Sometimes

BRAF variant status = Always Never Sometimes

TERT promoter variant status = Always Never Sometimes

MGMT methylation status = Always Never Sometimes

11. **Please specify circumstances for any molecular profiling performed 'sometimes'.**

______________________________________ ______________________________________

12. Please specify any molecular profiling performed routinely not listed above. ______________________________________

13. How do you / your MDT assess the extent of resection ascertained following initial surgery?

Immediate post-operative MRI scan (0-72hrs)

Immediate post-operative CT scan (0-72hrs)

Neurosurgeon's estimate

If other, please specify ______________________________________

Additional Comments

____

14. After initial surgery for low grade glioma, if indicated, what is your preferred timing for commencement of adjuvant radiotherapy?

Within 4 weeks Within 6 weeks

If other, please specify

____

Additional Comments

____

15. How confident are you with using and applying the RANO (Response Assessment in Neuro-Oncology) LGG (Low grade glioma) criteria?

Very unfamiliar Not confident Neutral

Confident

Very confident and comfortable and not confident

Additional Comments

___________________________________ ___________________________________

16. The phase III INDIGO study demonstrated improvements in progression free survival for selected patients with grade 2, IDH1/2 mutant oligodendroglioma or astrocytoma when treated with vorasidenib compared to observation.

Are you familiar with the INDIGO trial? Yes/No

What is your initial perception of the INDIGO data? ________________________

What proportion (%) of patients with a grade 2 glioma diagnosis would you consider suitable for treatment with vorasidenib?

____

What proportion (%) of patients with a grade 3 glioma diagnosis would you consider suitable for treatment with vorasidenib?

____

Assuming drug availability, do you have any concerns about initiating a suitable patient on vorasidenib?

____

Please answer the following questions based on this case information:

Case 1: 38-year-old female, gross total resection of an IDH-mutant grade 2 astrocytoma: No residual enhancing disease remaining on immediate post-operative MRI (<72hrs). Molecular profile: pending Clinically, asymptomatic (stable seizures, managed on levetiracetam), prompt surgical recovery.

17. Are there any specific molecular profiling results that you would request to guide treatment decisions? Yes No

If so, please specify

___________________________________ ___________________________________

What would be your recommended treatment approach for this case?

18. Observation alone Yes No

If so, what clinical and MRI interval would you institute?

___________________________________ ___________________________________

19. Adjuvant radiotherapy? Yes No

If yes, please specify which dose schedule will most likely be recommended (Please include Gy and number of fractions)

___________________________________ ___________________________________

20. Adjuvant chemotherapy? Yes No

If so, please specify choice of agents, number of cycles and whether concurrent or sequential chemotherapy would be recommended

___________________________________ ___________________________________

21. If surveillance alone is selected, what are the main factors that result in observation being chosen as most appropriate treatment? Rank answers on a scale of 1 to 5 (where 1 has the least influence and 5 has the most influence)

Patient preference

Volume of residual disease (<2cm)

Low symptomatic burden

Good performance status

Tumour molecular profile

22. **Are there other factors in addition to the above which would lead to your recommendation of surveillance alone?**

____

23. **Following initial surgery, what are the main indications for adjuvant radiotherapy?**

Rank answers on a scale of 1 to 5 (where 1 has the least influence and 5 has the most influence)

Patient preference

Volume of residual disease (<2cm)

Low symptomatic burden

Good performance status

Tumour molecular profile

24. **Are there any other factors (not listed above) that result in adjuvant radiotherapy being recommended?**

____

25. **Following initial surgery, what are the main indications for adjuvant chemotherapy?**

Rank answers on a scale of 1 to 5 (where 1 has the least influence and 5 has the most influence)

Patient preference

Volume of residual disease (<2cm)

Low symptomatic burden

Good performance status

Tumour molecular profile

26. **Are there any other factors (not listed above) that result in adjuvant chemotherapy being recommended?**

____

27. **If the original pathology had revealed a 1p/19q codeletion, confirming the diagnosis of a grade 2 oligodendroglioma, would your treatment recommendation change?**

Yes No

If yes, how?

____

28. If the original tumour pathology was found to harbour a CDKN2A/B homozygous deletion, would this alter your management recommendation?

Yes No

If yes, how?

_________________________________ _________________________________

29. Overall, what proportion of grade 2 astrocyoma cases at initial diagnosis would you expect to receive the following treatment recommendations? The sum of all entered values must be 100.

Observation alone

Radiotherapy alone

Chemotherapy alone

Combination of radiotherapy and chemotherapy

30. Overall, what proportion of grade 2 oligodendroglioma cases at initial diagnosis at your centre would you expect to receive the following treatment recommendations? The sum of all entered values must be 100.

Observation alone

Radiotherapy alone

Chemotherapy alone

Combination of radiotherapy and chemotherapy

31. If available, would you preference vorasidenib for this patient over current adjuvant therapies? Reminder: 38-year-old female, gross total resection of IDH-mutant grade 2 astrocytoma with no residual disease

Yes No

Additional Comments

_________________________________ _________________________________

If this same patient experienced tumour recurrence after 24 months, having had observation only, and underwent further resection, what would be your preferred salvage treatment?

32. Observation alone Yes No

If so, what clinical and MRI interval would you institute?

__________________________________ __________________________________

33. Adjuvant radiotherapy? Yes No

If yes, please specify which dose schedule will most likely be recommended (Please include Gy and number of fractions)

__________________________________ __________________________________

34. Adjuvant chemotherapy? Yes No

If so, please specify choice of agents, number of cycles and whether concurrent or sequential chemotherapy would be recommended

__________________________________ __________________________________

35. If vorasidenib was available at the time of this recurrence, would you preference this over the above standard salvage therapies?

Yes No

Additional Comments

____

Please answer the next questions based on the following case details: 58-year-old man with newly diagnosed grade 2 astrocytoma.

Case 2: Subtotal resection: non-enhancing rim of residual disease on post-operative MRI T2 FLAIR sequences. Clinically, recovering well postoperatively. Asyptomatic. Molecular profile: IDH1 mutant detected, 1p/19q codeletion NOT detected, TP53 variant detected, CDKN2A/B homozygous deletion NOT detected.

36. Are there any additional molecular profiling results you would like to guide your treatment recommendation? Yes No

If yes, please specify.

____

37. Observation alone? Yes No

If so, what clinical and MRI interval follow-up would you institute?

___________________________________ ___________________________________

38. Further surgery? Yes No

39. Adjuvant radiotherapy? Yes No

If yes, please specify what dose (Gy) and number of fractions that would be most likely recommended.

___________________________________ ___________________________________

40. Adjuvant chemotherapy? Yes No

If yes, please specify which agents, number of cycles and whether concurrent or sequential chemotherapy would be recommended

___________________________________ ___________________________________

41. **If available, and after a period of 12 months of monitoring only with stable disease, would you preference vorasidenib for this patient over current adjuvant therapies?**

Yes No

If so, why?

___________________________________ ___________________________________

42. If the original tumour pathology was found to harbour a CDKN2A/B homozygous deletion, would this alter your management?

Yes No

If yes, how?

____

43. If surveillance only was pursued initially and subsequent tumour recurrence occurred, what would be your treatment recommendation in the following circumstances?

Recurrence at >12 months post original resection, and vorasidenib is unavailable. ___________________________

Recurrence at >12 months post original resection, patient is asymptomatic, disease is resectable, vorasidenib is available but resection would preclude vorasidenib. ___________________________

Recurrence at >12 months in a symptomatic patient when vorasidenib is available, but surgery would preclude drug acccess.

____

Recurrence occurs at 6 months, and vorasidenib is available and unresctricted. Would you preference this over the established approach of salvage radiotherapy and/or chemotherapy? ___________________________

Please answer the next questions based on the following case details:

Case 3: 58yo female with newly diagnosed IDH-mutant grade 3 oligodendroglioma. Near gross total resection with both enhancing and non-enhancing residual disease on post-operative MRI. Symptomatic. Molecular profile: IDH1 variant detected, TERT promoter variant detected, 1p/19q codeletion detected.

What would be your recommended treatment approach for this case?

44. Observation alone? Yes No

If so, what clinical and MRI interval follow-up would you institute?

___________________________________ ___________________________________

45. Further surgery? Yes No

46. Adjuvant radiotherapy? Yes No

If yes, please specify what dose (Gy) and number of fractions that would be most likely recommended.

___________________________________ ___________________________________

47. Adjuvant chemotherapy? Yes No

If yes, please specify which agents, number of cycles and whether concurrent or sequential chemotherapy would be recommended

___________________________________ ___________________________________

48. Overall, what proportion of grade 3 astrocytoma cases would you expect to receive the following treatments at initial diagnosis? The sum of all entered values must be 100.

Observation alone

Radiotherapy alone

Chemotherapy alone

Combination of radiotherapy and chemotherapy

49. Overall, what proportion of grade 3 oligodendroglioma cases at initial diagnosis would you expect to receive the following treatment recommendations: The sum of all entered values must be 100.

Observation alone

Radiotherapy alone

Chemotherapy alone

Combination of radiotherapy and chemotherapy

50. Thank you for your participation in this patterns of care survey. Once analysed, results will be presented at relevant scientific forums. Please add any additional comments or feedback below. ___________________________________

File S2. Genomic and Molecular biomarkers tested

- IDH1 (R132H only)
- IDH1 and IDH2
- 1p/19q codeletion
- CDKN2A/B homozygous deletion
- BRAF variant status
- H3 K27 status
- TERT promotor variant status
- EGFR amplification
- MGMT methylation status
